# A National Cross-Sectional Study of the Characteristics, Strengths, and Challenges of College Students With Attention Deficit Hyperactivity Disorder

**DOI:** 10.7759/cureus.21520

**Published:** 2022-01-23

**Authors:** Emily Hotez, Kashia A Rosenau, Priyanka Fernandes, Kevin Eagan, Lindsay Shea, Alice A Kuo

**Affiliations:** 1 General Internal Medicine, David Geffen School of Medicine, The University of California, Los Angeles (UCLA), Los Angeles, USA; 2 Medicine, David Geffen School of Medicine, The University of California, Los Angeles (UCLA), Los Angeles, USA; 3 Division of Medicine-Pediatrics, David Geffen School of Medicine, The University of California, Los Angeles (UCLA), Los Angeles, USA; 4 Education, The University of California, Los Angeles (UCLA), Los Angeles, USA; 5 Public Health, A.J. Drexel Autism Institute, Drexel University College of Medicine, Philadelphia, USA

**Keywords:** united states, higher education, college, emerging adulthood, adhd

## Abstract

Introduction: A substantial proportion of college students experience challenges transitioning from pediatrics to the adult healthcare system. Combined internal medicine and pediatrics (Med-Peds) providers are frequently tasked with facilitating this transition and promoting the health and well-being of this population. There is an increasing proportion of college students with Attention Deficit Hyperactivity Disorder (ADHD) in the U.S. This population experiences particularly pronounced challenges navigating the healthcare system and, as a result, often contends with fragmented healthcare. These issues are due to a range of factors, including lack of physician training, education, and resources, as well as a dearth of available research that can inform Med-Peds providers' efforts to support college students with ADHD.

Methods: The current study compared a nationally representative sample of U.S. college freshmen with ADHD to those without ADHD on health, academic, and non-academic capacities. This study analyzed population-weighted data from the Cooperative Institutional Research Program’s Freshman Survey.

Results: Students with ADHD were more likely to report co-occurring conditions and feelings of depression and overwhelm. They were less likely to report emotional health that was above average or in the highest 10^th^ percentile. Although they reported lower overall academic aspirations, they were more likely to rate themselves in the highest 10th percentile on a range of non-academic capacities.

Conclusion: The results from this study can inform efforts among Med-Peds providers seeking to promote the health and well-being of college students with ADHD.

## Introduction

Emerging adulthood (ages 18 to 30 years) coincides with the transition from pediatrics to adult healthcare. Combined internal medicine and pediatrics (Med-Peds) providers are tasked with promoting the health and well-being of this population during this transition. Given their role in supporting emerging adults, Med-Peds providers are often confronted with the need to support college students.

There is an increasing proportion of college students with attention deficit hyperactivity disorder (ADHD), a developmental condition characterized by inattention, hyperactivity, and/or impulsivity and experienced by at least 4% of the U.S. adult population [1]. The majority of previous research on college students with ADHD, however, is reliant on small, non-representative samples [2]. Further, the available research does not capture a sufficiently broad range of social, psychological, and developmental capacities [2]. These limitations impede the extent to which research is generalizable and translatable to Med-Peds providers' clinical practices. Nationally representative research that captures the characteristics, strengths, and challenges of college students with ADHD would inform Med-Peds providers’ efforts to provide quality healthcare for this population.

The unmet needs of college students with ADHD

The available research on college students with ADHD suggests that they have a range of unmet needs [3,4]. Of particular relevance to Med-Peds providers is that adults with ADHD experience a 12.7-year reduction in life expectancy relative to their counterparts without ADHD [3]. This disparity is partially due to health issues while transitioning to adulthood [4]. Indeed, relative to their counterparts without ADHD, emerging adults with ADHD disproportionately struggle with implementing health-based lifestyle behaviors, engage in problematic substance use, and experience psychological distress and social maladjustment [4]. The challenges presented in the college transition may exacerbate these issues [5], in part by increasing exposure to discrimination and internalized stigma [6-8] and triggering concealment and camouflaging [6,9-11]. Thus, college students with ADHD represent a particularly vulnerable population concerning health and well-being.

This heightened vulnerability is exacerbated by the unmet academic needs of college students with ADHD. Indeed, they experience lower overall achievement [12], including lower test scores and grade point averages [13], difficulties in reading [12], and other academic challenges [14]. To be sure, academic difficulties are often the reason for a child’s initial referral for clinical evaluation for ADHD [15]. The achievement gap in college, however, cannot be attributed solely to individual characteristics. Rather, numerous barriers in the higher education environment compromise the success of diverse learners [16]. For example, students with ADHD may benefit from distinct learning and studying approaches that may not be aligned with those that are traditionally promoted in the college environment [17]. These difficulties have important implications for success during and post-college [18].

The majority of previous research focuses on these challenges. Individuals with ADHD, however, may possess exceptional skills related to problem-solving, entrepreneurial endeavors, and other areas [19,20]. As a result of limited research on strengths, providers are tasked with continuously reacting to patients' problems rather than proactively promoting their thriving and well-being. Indeed, a strengths-based approach would elucidate opportunities for Med-Peds providers to fully support this population.

The current study

A lack of robust research on college students with ADHD perpetuates shortcomings in healthcare practice and policy for this population [21,22]. It is particularly important to recognize these co-occurring challenges in the aftermath of the COVID-19 pandemic, during which all college students experienced significant health, financial, educational, and professional obstacles [23]. The current study addresses gaps in the field by comparing a nationally representative U.S. sample of first-year college students with ADHD to students without ADHD on demographics, mental and physical health, academic achievement, and confidence in their academic and social capacities, with the broader goal of promoting the health and well-being of this population.

## Materials and methods

The data for this cross-sectional study come from five separate administrations of the Cooperative Institutional Research Program (CIRP) Freshman Survey, which collects snapshot data on a nationally representative sample of incoming first-year college students in the U.S. Most campuses conduct the survey during orientation, wherein about one hour for survey administration is allowed. Since its inaugural administration in 1966, more than 15 million students attending more than 1100 institutions have completed the survey. The current study is approved by the Higher Education Research Institute, UCLA Graduate School of Education Institutional Review Board (#10-001293).

Sample

The analytic sample included first-year, full-time students enrolled at four-year non-profit colleges and universities who completed the CIRP Freshman Survey in 2012, 2014, 2016, 2018, or 2019. Students with ADHD were identified by self-identification through the question, “Do you have any of the following disabilities or medical conditions?” There were seven response options, including ADHD. Population weights were calculated for each survey cohort by considering representation by students’ sex, race or ethnicity, and several characteristics of the institutions they attended, including control (i.e., public, private), type (i.e., university, four-year college), and selectivity level (i.e., average Scholastic aptitude test [SAT] Math and SAT Reading scores) [24]. When applied, the weight-adjusted the sample to represent all first-year, full-time, first-year students entering four-year non-profit colleges and universities in the U.S. in 2012, 2014, 2016, 2018, and 2019.

Data analysis

The analyses compared first-year full-time self-identified students with ADHD with their peers who did not report ADHD on a select set of demographic characteristics (e.g., race/ethnicity), emotional and mental health indicators (e.g., feelings of depression and overwhelm), measures of academic achievement (e.g., SAT scores), and items related to confidence in their academics (e.g., self-rated academic ability) and social skills (e.g., self-rated understanding of others).

Many of the survey items appear as Likert scales, and the analyses for those items typically relied on chi-square statistics to determine whether the ADHD sample significantly differed from the comparison group in the proportion of respondents choosing the same value (e.g., frequently, above-average/highest 10%). For other categorical variables (e.g., race/ethnicity, sex, parental education), the analyses compared the proportions in each category between the two samples and relied on chi-square statistics to indicate statistically significant differences. Continuous measures (e.g., standardized test scores, latent construct scores) relied upon t-tests to determine significant differences between the sample means for each group. Of note, because of changes in the SAT scoring system in 2016, we have presented the SAT Math and SAT Reading scores in all years and did not include the SAT Writing scores in our analyses.

Several latent constructs were calculated from individual items using item response theory (IRT), which represents a model-based approach for psychometric data analysis. It offers several advantages over traditional factor analysis, which is based on classical test theory. A full discussion of IRT and the corresponding item weights for all latent constructs have been published previously in a technical report [25]. Additionally, effect sizes were calculated on the three latent constructs: 0.20 was considered to be a small, 0.50 a medium, and 0.80 a large effect size.

The analyses used list-wise deletion for cases with missing data. Due to the high probability of obtaining significant p-values in analyses with large samples, percentage differences of 3% or greater were considered practically significant. All tests were performed at a two-tailed alpha level of 0.01. All analyses were completed in IBM Corp. Released 2021. IBM SPSS Statistics for Windows, Version 28.0. Armonk, NY: IBM Corp.

## Results

With the population weights applied, the total sample was 7,73,1640, with 435,234 students with ADHD (5.6%) and 7,296,405 students without ADHD. ADHD subtypes were not assessed in the current study.

Demographic differences are displayed in Table 1. Relative to the comparison group, significantly fewer college students with ADHD were female (ADHD: 43.1%; comparison: 55.3%), Asian (ADHD: 3.7%; comparison: 10.2%), Black/African American (ADHD: 6.4%; comparison: 9.6%), or Latinx (ADHD: 4.0%; comparison: 10.6%), p < 0.001. Students with ADHD were significantly more likely to be White (ADHD: 70.7%; comparison: 55.4%) and less likely to have parents who completed a high school diploma or less (ADHD: 10.5%; comparison: 18.1%), p < 0.001. Significantly more students with ADHD reported that their parents’ highest level of education was a graduate school (ADHD: 44.4%; comparison: 32.8%), p < 0.001. Students with ADHD were significantly less likely to report an estimated family annual income of less than $30,000 (ADHD: 12.8%; comparison: 17.3%) and significantly more likely to report an annual income of $250,000 and higher (ADHD: 16.8%; comparison: 9.4%), p < 0.001.

**Table 1:** Demographic information of first-year full-time college students stratified by self-reported ADHD ADHD: attention deficit hyperactivity disorder

	Comparison Group (%)	ADHD (%)	p value
Characteristics			
Gender			
Female	55.3%	43.1%	< 0.001
Age			
Under 18	1.3%	1.0%	<0.004
18	69.8%	65.0%	< 0.001
19	26.7%	31.2%	< 0.001
20	1.1%	1.6%	< 0.001
21 or older	1.1%	1.1%	
Race/Ethnicity			
Native American/American Indian	0.3%	0.2%	
Asian	10.2%	3.7%	< 0.001
Black/African American	9.6%	6.4%	< 0.001
Latino	10.6%	4.0%	< 0.001
White	55.4%	70.7%	< 0.001
Other	1.2%	1.0%	< 0.001
Multi-racial	12.7%	14.0%	
Parent/Guardian Education			
HS diploma or less	18.1%	10.5%	< 0.001
Bachelor’s degree	33.2%	33.5%	
Some college	15.9%	11.7%	< 0.001
Graduate school	32.8%	44.4%	< 0.001
Income (Mean, SD)			
Under $30,000	17.2%	12.8%	< 0.001
$30,000 - $59,999	17.3%	12.8%	< 0.001
$60,000 - $99,999	22.3%	20.8%	< 0.001
$10,000 - $149,999	19.7%	19.8%	
$150,000-$249,999	14.2%	17.0%	< 0.001
$250,000 and higher	9.4%	16.8%	< 0.001

Health differences are displayed in Table 2. Significantly fewer students with ADHD reported emotional health that was above average or in the highest 10% bracket (ADHD: 14.1%; comparison: 17.5%), p < 0.001. Significantly more students with ADHD reported feelings of depression and overwhelm in the past year (ADHD: 44.9%; comparison: 36.9%), p < 0.001. Significantly more students with ADHD also reported having other co-occurring conditions, including learning disabilities (ADHD: 20.0%; comparison: 2.3%), Autism spectrum disorder (ADHD: 4.5%; comparison: 0.5%), psychological disorders (ADHD: 31.2%; comparison: 8.7%), physical disabilities (ADHD: 8.9%; comparison: 3.8%), chronic illness (ADHD: 4.8%; comparison: 2.3%), and other disabilities (ADHD: 10.5%; comparison: 4.4%), p < 0.001.

**Table 2:** Health characteristics of first-year full-time college students stratified by self-reported ADHD ADHD: attention deficit hyperactivity disorder

	Comparison Group (%)	ADHD (%)	p value
Characteristics			
Physical health			
Above average/highest 10%	18.4%	18.5%	< 0.001
Emotional health			
Above average/highest 10%	17.5%	14.1%	< 0.001
Frequent feelings of (in past year)			
Depression	11%	21.6%	< 0.001
Being overwhelmed by all s/he had to do	36.9%	44.9%	< 0.001
Disabilities or chronic illnesses			
Learning disability	2.3%	20.0%	< 0.001
Autism spectrum disorder	0.5%	4.5%	< 0.001
Psychological disorder	8.7%	31.2%	< 0.001
Physical disability	3.8%	8.9%	< 0.001
Chronic illness	2.3%	4.8%	< 0.001
Other disability	4.4%	10.5%	< 0.001

Differences in academic aspirations and achievement are displayed in Table 3. Significantly more students with ADHD reported that obtaining a bachelor’s degree was their career aspiration (ADHD: 27.2%; comparison: 23.7%) and significantly fewer reported aspirations for a medical or health-related degree (ADHD: 4.7%; comparison: 6.7%), p < 0.001. Significantly fewer students with ADHD reported an average grade in high school of A-, A, or A+ (e.g., A+, ADHD: 12.9%; comparison: 28.9%) and significantly more reported average grades of B+, B, B-, or C+ (e.g., C+, ADHD: 5.0%; comparison: 2.0%), p < 0.001. Statistically significant differences between students with and without ADHD also emerged across all average standardized test scores. Students with ADHD scored, on average, 10 points lower than their counterparts on SAT Math, p < 0.001.

**Table 3:** Academic characteristics of first-year full-time college students stratified by self-reported ADHD ADHD: attention deficit hyperactivity disorder, SAT: Scholastic aptitude test, SD: standard deviation

	Comparison Group (%)	ADHD (%)	p value
Characteristics			
Degree aspirations			
None	0.6%	1.1%	< 0.001
Vocational certificate	0.1%	0.3%	< 0.001
Associate degree	0.6%	1.1%	< 0.001
Bachelor’s degree	23.7%	27.2%	< 0.001
Master’s degree	23.4%	25.3%	< 0.001
Law degree	2.5%	2.7%	
Medical/health-related degree	6.7%	4.7%	< 0.001
Doctor of Philosophy	6.8%	6.8%	
Professional doctorate	3.5%	3.4%	
Other	0.9%	1.4%	< 0.001
Average grade in high school			
A or A+	28.9%	12.9%	< 0.001
A-	27.2%	21.9%	< 0.001
B+	19.8%	22.7%	< 0.001
B	16.5%	24.7%	< 0.001
B-	4.9%	10.5%	< 0.001
C+	2.0%	5.0%	< 0.001
C	0.8%	2.2%	< 0.001
D	0.0%	0.1%	< 0.001
Standardized test scores (Mean, SD)			
American College Testing (ACT) composite	25.4 (5.8)	25.1 (6.2)	< 0.001
SAT Math	597.1 (110.8)	585.1 (110.8)	< 0.001
SAT Reading	590.7 (101.0)	589.3 (107.7)	< 0.001
Number of college applications submitted (Mean, SD)	5.4 (2.9)	5.2 (2.9)	< 0.001

Differences in academic and non-academic capacities are displayed in Table 4. Significantly more students with ADHD rated themselves in the highest 10 percent on artistic ability (ADHD: 11.7%; comparison: 7.4%), computer skills (ADHD: 8.6%; comparison: 6.3%), creativity (ADHD: 25.5%; comparison: 16.2%), public-speaking ability (ADHD: 15.9%; comparison: 12.4%), social self-confidence (ADHD: 18.6%; comparison: 15.5%), self-understanding (ADHD: 23.1%; comparison: 19.5%), understanding of others (ADHD: 29.1%; comparison: 25.7%), compassion (ADHD: 31.3%; comparison: 27.3%), and risk-taking (ADHD: 19.2%; comparison: 13.3%). Significantly fewer students with ADHD rated themselves in the highest 10 percent on academic ability (ADHD: 14.9%; comparison: 20.1%) and drive to achieve (ADHD: 29.2%; comparison: 35.6%).

**Table 4:** Self-reported academic and non-academic characteristics of first-year full-time college students stratified by self-reported ADHD ADHD: attention deficit hyperactivity disorder

	Comparison Group (%)	ADHD (%)	p value
Characteristics			
Self-Rated, Highest 10%			
Academic ability	20.1%	14.9%	< 0.001
Artistic ability	7.4%	11.7%	< 0.001
Competitiveness	22.2%	24.3%	< 0.001
Computer skills	6.3%	8.6%	< 0.001
Cooperativeness	21.7%	20.4%	< 0.001
Creativity	16.2%	25.5%	< 0.001
Drive to achieve	35.6%	29.2%	< 0.001
Leadership ability	23.5%	24.9%	< 0.001
Mathematical ability	13.7%	12.1%	< 0.001
Public speaking ability	12.4%	15.9%	< 0.001
Intellectual self-confidence	18.9%	21.0%	< 0.001
Social self-confidence	15.5%	18.6%	< 0.001
Self-understanding	19.5%	23.1%	< 0.001
Spirituality	12.4%	12.5%	
Understanding of others	25.7%	29.1%	< 0.001
Writing ability	12.2%	15.1%	< 0.001
Compassion	27.3%	31.3%	< 0.001
Risk-taking	13.3%	19.2%	< 0.001

## Discussion

The current study represents one of the only nationally representative studies to report the characteristics, strengths, and challenges of freshmen with ADHD entering four-year colleges and universities in the U.S. This research identified several key differences between college students with ADHD and their counterparts on demographic characteristics, health outcomes, and a range of academic and non-academic strengths and challenges. Findings from this research have the potential to inform Med-Peds providers’ efforts to support their diverse emerging adult patients.

First, 5.6% of U.S. college students reported having ADHD. This is slightly higher than the reported U.S. prevalence of 4%. This discrepancy may be due to differences between college students and the general adult population and/or differences between ADHD self-identification and diagnosis [26]. It may also be due to changes to the diagnostic and statistical manual of mental disorders (DSM-5) that have resulted in increased ADHD reporting in recent years [27]. There is also evidence for late ADHD diagnosis that may contribute to these differences [28].

Consistent with childhood data reported in the National Survey of Children’s Health and the Early Childhood Longitudinal Study [29,30], students with ADHD were significantly more likely to be male, white, and have parents with greater educational attainment. The under-diagnosis of racial and ethnic minority children with ADHD is well-documented [31,32]. Our study suggests that these disparities in childhood diagnoses translate to disparities in self-identification in adulthood. As a result, there may be some students who do not self-identify with an ADHD diagnosis, but experience ADHD-specific challenges in the transition to the college environment, particularly females and racial and ethnic minorities. This finding underscores the need for Med-Peds providers to pay particular attention to emerging adults who may experience challenges consistent with an ADHD diagnosis but may not identify as having ADHD [33].

Also consistent with previous research [34], significantly more students with ADHD reported having other co-occurring developmental conditions and health challenges, including learning disabilities, autism spectrum disorder, psychological disorders, physical disabilities, chronic illnesses, and other disabilities. In addition, significantly fewer students with ADHD reported emotional health that was above average or in the highest 10% bracket relative to their counterparts. Further, significantly more students with ADHD reported feelings of depression and getting overwhelmed in the past year relative to their counterparts.

The mental health challenges identified in this research may be partially attributed to findings from previous research that suggest that college students with disabilities experience discrimination, stigma, and/or marginalization [6]. Given that students with ADHD were disproportionately likely to have multiple disabilities (e.g., autism), experiences of stigma may be compounded for this population. Taking into account these findings, Med-Peds providers should consider the broad range of challenges that students with ADHD may face beyond those traditionally associated with ADHD (e.g., learning, attention, etc.). Efforts to support these patients should include providing access to a range of educational, health, and social resources. Indeed, there is a well-established urgent need to support college students’ mental health to counteract the detrimental impacts of the COVID-19 pandemic [23]; our study suggests students with ADHD may have even more pronounced support needs.

We also found significant differences concerning academic characteristics. A higher proportion of students with ADHD reported that obtaining a bachelor’s degree was their career aspiration, and a lower proportion rated themselves in the highest 10 percent on academic ability and drive to achieve. In addition to these findings, fewer students with ADHD reported aspirations for a medical or health-related degree. These findings suggest that higher education is not sufficiently promoting student motivation and drive to achieve among diverse learners upon college entry. Research shows that "growth-oriented" classrooms may be beneficial for enhancing the motivation and success of students with ADHD [35]. In practice, this involves instructors structuring educational environments so that students strive to reach their own individualized goals [35]. In concern with reforms to higher education, Med-Peds providers can encourage their college student patients to engage in self-reflection and goal-setting in and out of the classroom.

Finally, students with ADHD reported several strengths. Significantly more students with ADHD rated themselves in the highest 10 percentile on artistic ability, computer skills, creativity, public-speaking ability, social self-confidence, self-understanding, understanding of others, compassion, and risk-taking relative to their counterparts. This is consistent with previous research that has found college students with ADHD to be more likely to have entrepreneurial intentions [36].

The finding that college students with ADHD have different strengths relative to their counterparts without ADHD suggests several next steps for higher education and healthcare. In education, the findings lend support to the importance of integrating principles of Universal Design in college classrooms. Universal Design is an educational approach that ensures that all types of learners with different strengths have opportunities to display their abilities and knowledge. Concerning healthcare, it would be beneficial for Med-Peds providers to support their college student patients in cultivating self-determination and self-advocacy. During appointments, this might involve empowering patients to engage with support networks in college (e.g., peers and faculty) through both virtual and in-person settings; providing suggestions for them to employ a range of studying and learning skills, and supporting them in pursuing opportunities that can leverage their unique strengths.

The current study had several limitations. First, we relied on self-identification to determine ADHD status. Future research should seek to integrate more robust diagnostic measures that include ADHD subtypes. Second, our study was cross-sectional. Research is needed to investigate students with ADHD over time. Finally, this study reported data from a large, national survey. Future studies with qualitative methodologies can add additional insight and depth to these findings.

## Conclusions

Findings from the current study provide key insights into college students with ADHD in the U.S. In describing the characteristics, strengths, and challenges of this population, these findings illuminate opportunities for Med-Peds providers to more effectively promote their health and well-being during and after college. In particular, the current study sheds light on targeted areas that require support (e.g., mental health) and can be further cultivated (e.g., non-academic abilities) to promote the overall health and well-being of college students with ADHD. These next steps are particularly important in the aftermath of the COVID-19 pandemic, which resulted in myriad challenges for college students. Future studies should continue to investigate opportunities to support college students with ADHD.
